# Classification of Mammographic ROI for Microcalcification Detection Using Multifractal Approach

**DOI:** 10.1007/s10278-022-00677-w

**Published:** 2022-07-19

**Authors:** Nadia Kermouni Serradj, Mahammed Messadi, Sihem Lazzouni

**Affiliations:** Biomedical Engineering Laboratory, Faculty of Technology, Abou Bekr Belkaid University, 13000 Tlemcen, Algeria

**Keywords:** Multifractal, Multifractal spectrum, Mammogram pre-processing, Microcalcification detection, Multifractal features and ROI classification

## Abstract

Microcalcifications (MCs) are the main signs of precancerous cells. The development of aided-system for their detection has become a challenge for researchers in this field. In this paper, we propose a system for MCs detection based on the multifractal approach that classifies mammographic ROIs into normal (healthy) or abnormal ROIs containing MCs. The proposed method is divided into four main steps: a mammogram pre-processing step based on breast selection, breast density reduction using haze removal algorithm and contrast enhancement using multifractal measures. The second step consists of extracting the normal and abnormal ROIs and calculating the multifractal spectrum of each ROI. The next step represents the extraction of the multifractal features from the multifractal spectrum and the GLCM characteristics of each ROI. The last step is the classification of ROIs where three classifiers are tested (KNN, DT, and SVM). The system is evaluated on images from the INbreast database (308 images) with a total of 2688 extracted ROIs (1344 normal, 1344 with MC) from different BI-RADS classes. In this study, the SVM classifier gave the best classification results with a sensitivity, specificity, and precision of 98.66%, 97.77%, and 98.20% respectively. These results are very satisfactory and remarkable compared to the literature.

## Introduction

More than two million women in the world are affected by breast cancer and more than five hundred thousand women die each year of this disease [1]. Awareness, screening and early detection remain the only ones to fight against this scourge. Mammography is the most effective X-ray imaging exam and used technique for breast cancer detection. However, the complex architecture of the breast, the distribution of breast density, the image quality, and the level of experience of the radiologist are all factors that influence the reading of the mammogram and its analysis where the radiologist can miss the small abnormal details present in the mammogram such as microcalcifications (MCs) which are calcium deposits representing the main signs of precancerous cells. Hence, great efforts of scientific researchers to develop computer-aided detection or diagnosis (CAD) systems aimed at assisting radiologists in the detection of abnormalities and the diagnosis of breast cancer. These systems increase the cancer detection rate at an early stage and reduce false positive interpretations.

The classification of mammographic region of interest (ROI) has been the subject of several scientific studies and has become a challenge for researchers in this field given the importance and considerable assistance of radiologists in the early detection of breast cancer [2]. Most of the proposed CAD systems for the classification of mammographic ROI go through four major stages:

- *Pre-processing*: This stage consists of removing noise and artifacts from the mammogram, selecting the breast, removing the pectoral muscle and the background, and, finally, enhancing the contrast. Many techniques for mammogram enhancement have been proposed in the literature. Table 1 summarizes all these techniques [3].
- *ROI extraction*: This is done either by cropping (with the help of the ground truth or the annotation of the expert) or by segmentation approaches. Different ROI sizes were considered in different papers.
- *Feature extraction*: Different sets of features are computed in the literature: statistical features (mean, standard deviation, skewness, uniformity, kurtosis, smoothness…), textural features using gray-level co-occurrence matrix (GLCM) or gray-level run length matrix (GLRLM) (contrast, homogeneity, correlation, entropy, sum average, sum entropy, difference variance, difference entropy, inverse difference moments…), shape features (area, perimeter, convexity, circularity, rectangularity, compactness, area ratio, perimeter ratio…), and multiscale features (wavelets coefficients, directional sub-bands coefficients…).
- *Classification*: Extracted features are fed to a machine-learning algorithm to classify the ROI (normal/abnormal, benign/malignant). A large number of machine learning algorithms have been proposed in the literature, and the most frequently used models are support vector machines (SVM) and artificial neural networks (ANN) [2].

**Table 1:** Mammogram enhancement techniques in the literature [3]

Technique	Methods
Contrast stretching	• *Adaptive neighborhood processing* - Fixed neighborhood approaches - Adaptive neighborhood contrast enhancement - Gradient and local statistics based enhancement • *Histogram-based enhancement techniques* - Histogram equalization - Adaptive histogram equalization (AHE) - Contrast limited adaptive histogram equalization (CLAHE) - Histogram modified local contrast enhancement - Fuzzy clipped CLAHE (FC-CLAHE) • *Unsharp masking (UM)-based enhancement techniques* - Linear and order statistics UM - Quadratic UM - Rational, cubic, and adaptive UM - UM based on region segmentation - Non-linear UM
Region based and Feature based techniques	• *Region-based enhancement techniques* - Region growing algorithm - Region based algorithm using watershed segmentation - Direct image contrast enhancement algorithm • *Feature-based enhancement techniques* - Wavelet-based multi-resolution techniques - Laplacian pyramid-based techniques - Miscellaneous multi-resolution techniques
Non-linear techniques	• Morphological filtering • Fuzzy-based enhancement techniques • Enhancement using non-linear filtering

However, we also find in the literature some CAD systems that use the whole mammogram to detect the suspicious region without going through the stage of ROI extraction. Some other CAD systems do not go through the pre-processing stage. Other CAD systems initially extract a large number of features, then go through the stage of relevant feature selection.

Moreover, different issues have been studied in the classification of mammographic ROIs. We find the classification of normal and abnormal ROIs and the classification of benign and malignant ROIs where suspicious ROIs are either masses [4–7], or MCs [8–11], or without specifying the type of lesion [12–18].

In this work, we are interested in the detection of ROIs containing MCs, in other words, classifying ROIs into normal or abnormal ROI (containing MCs). Therefore, we present some recent works that have worked on the same problem. In [19], authors proposed a system based on wavelet transform for classification of normal and abnormal ROI of MCs, then the classification of benign and malignant MCs. Without pre-processing step, they directly worked on the cropped ROI where they extracted statistical and multi-scale features based on the Haar wavelet transform, interest points, and corners. In total, 50 features were fed to the random forest classifier. The system was tested on the BCDR data base with a total of 192 ROI (96 normal, 96 abnormal). Obtained accuracy, sensitivity, and specificity of classification were 95.83%, 96.84%, and 95.09% respectively. In [20], authors proposed a system based on pattern recognition and size prediction of MCs. The pattern of a MC was found based on its physical characteristics (the reflection coefficient and mass density of the lesion). Then, the detected MC pattern is projected as a 3D image to find the size of the MC. This was tested on 100 images from the DDSM database (100 abnormal, 10 normal). Obtained accuracy, sensitivity, and specificity of classification were 99%, 99%, and 100% respectively. In [21], authors compared three feature extraction techniques: the rotation invariant local frequency (RILF), the local binary pattern (LBP), and segmented fractal texture analysis (SFTA) where the RILF technique gave the best results. The IRMA database was used with a total of 1620 ROIs (932 normal, 688 abnormal). Extracted histogram features were fed to the SVM classifier. Obtained accuracy, sensitivity, and specificity of classification were 91.10%, 98.04%, and 81.17% respectively. In [22], authors proposed a system based on transfer learning for the classification of normal and abnormal ROI containing MCs or masses. They started with a pre-processing step where they used morphological operations, binarization, and component selection. ROIs were cropped and resized. Features were extracted and classified using ResNet. The system was tested on the Mini-Mias database, and it achieved an accuracy of 95.91%. In [23], authors proposed a system for classification of normal and abnormal ROI with MCs, then the classification of benign and malignant MCs. They started by ROIs extraction which are pre-processed using the pixel assignment-based spatial filter to enhance the visibility of MCs in mammograms. Then, six statistical features were extracted and fed to the SVM, multilayer perceptron neural network (MLPNN), and linear discriminant analysis (LDA) classifiers. This was tested on 219 mammograms extracted from the DDSM data base (124 normal, 95 abnormal). Obtained accuracy, sensitivity, and specificity were 90.9%, 98.4%, and 81.3% respectively. In [24], authors proposed an improvement of the previous work [19] by using only two features (interest point and interest corners) with a total of 260 ROIs (130 normal, 130 abnormal). Obtained accuracy, sensitivity, and specificity were 97.31%, 94.62%, and 100% respectively using random forest classifier.

In this paper, we propose a novel approach to the classification of normal and abnormal ROI containing MCs based on multifractal analysis. First, the original mammogram is pre-processed using the proposed approach in [25] for mammogram enhancement which is based on multifractal measures. Then, the ROI is cropped automatically according to the expert annotation. For each ROI (normal and abnormal), the multifractal spectrum is computed using two different methods and five features are extracted. Finally, multifractal features combined with GLCM features are fed to three classifiers (SVM, KNN and DT) for the classification of normal and abnormal ROIs.

The paper is organized as follows: a section of “Materials and methods” presents the multifractal theory and the proposed approach; the “Results and discussion” section discusses the obtained results; and, finally, we end with the “Conclusion” section.

## Materials and Methods

### Fractal and Multifractal Theories

The mathematician Benoît Mandelbrot introduced the concept of fractals to describe complex objects whose Euclidian geometry did not allow their description. These objects are characterized by the properties of scale invariance, also called self-similarity where similar structures are viewed on all scales, i.e., the object is composed of smaller parts, where each part is a smaller copy of the whole. The main parameter used to describe the geometry and heterogeneity of these irregular objects and quantify their internal structure repeated over a range of scales is the fractal dimension (${D}_{F}$). For a fractal object, the number of features of a certain size $\varepsilon$, $N(\varepsilon )$, varies as [26]:

1 $$N\left(\varepsilon \right)\sim {\varepsilon }^{-{D}_{F}}$$

Equation (1) is the scaling (or power) law that describes the size distribution of the object.

One of the simplest ways to calculate the fractal dimension ${D}_{F}$ is to calculate the logarithmic ratio of change in detail ($N$) to change in scale ($S$): ${D}_{F} = (log N)/(log S)$. The most used method to estimate the ${D}_{F}$ is the box-counting technique. This method uses sample elements, which are an array of pixel points. The scale $S$ relates to the sampling element’s size. It consists of covering a measure with boxes of size $L$ and counting the number of boxes containing at least one pixel representing the object under study $N(L)$, ${D}_{F}$ is estimated as [26]:

2 $${D}_{F}= \underset{L\to 0}{\mathit{lim}}\frac{\mathit{log}N(L)}{log(\frac{1}{L})}$$

Using Eq. (2), the box-counting dimension ${D}_{F}$ can be determined as the negative slope of $log N(L)$ versus $log(L)$ measured over a range of boxes sizes.

However, the limits of monofractal analysis remain in its inability to describe the local fractal behaviors of images, hence the birth of multifractal analysis.

Multifractals are a generalization of fractals. A multifractal object is more complex and it is always invariant by translation. Multifractal analysis studies the global regularity from the local regularity of the signal. It is based on the estimation of two sets of coefficients: the Hölder exponents that quantifies the local regularity of the signal and the multifractal spectrum that quantifies the multifractality of the signal. It consists of decomposing the signal into subsets having the same regularity, and then measuring the “size” of the subsets thus obtained. The local regularity of a signal $X$ at any point $t$ is determined by the Hölder exponent ${\alpha }_{X}\left(t\right)$ defined as [27]:

3 $${\alpha }_{X}\left(t\right)=\underset{v\to t}{\mathit{lim}}\mathit{inf}\frac{log|X\left(v\right)-X(t)|}{log|v-t|}$$

Since the exponent ${\alpha }_{X}$ is defined in all $t$, the original signal $X$ can be associated with its Hölder function, $t \to {\alpha }_{X} (t)$, which describes how the regularity of $X$ varies: the smaller ${\alpha }_{X}\left(t\right)$, the more irregular $X$ is in $t$, and vice versa [27].

The second step in multifractal analysis is to study the sets [27]:

4 $${E}_{\alpha }=\{t:{\alpha }_{X}\left(t\right)=h\}$$

where ${E}_{\alpha }$ is the sets of points (pixel locations for 2D signal) of the same regularity. The measurement of these sets gives a global description of the singularities distribution of the signal $X$. This can be done using a geometrical or statistical approach. The result is, in all cases, a “multifractal spectrum,” i.e., a function $\alpha \to f(\alpha )$, which describes “how many” points of the signal have a regularity equal to $\alpha$. In the geometrical approach, the multifractal spectrum represents the dimension of all the points having the $\alpha$ exponent, i.e., ${E}_{\alpha }$. In the statistical approach, the multifractal spectrum is estimated from the probability of encountering a pixel whose regularity is in the order of $\alpha$ [27]. In this paper, we use the statistical approach.

Several methods for estimating the multifractal spectrum have been proposed in the literature. There are methods based on box counting, methods based on wavelets and methods based on detrended fluctuation analysis (DFA). A summary of these methods is presented in Table 2. In this paper, we use the generalized fractal dimensions method and the moving average DFA method for the classification of mammographic ROI.

**Table 2:** Multifractal spectrum estimation methods for two dimensional signal

Box-counting methods	- Generalized fractal dimensions [28] - The “sand box” or cumulative mass method [29] - The large-deviation multifractal spectrum [30]
Wavelet methods	- The wavelet transform modulus maxima (WTMM) method [31] - The wavelet leaders method [32]
Detrending analysis methods	- Multifractal detrended fluctuation analysis (MFDFA) [33] - Multifractal detrended moving average (MFDMA) [33] - Multiscale multifractal detrended-fluctuation analysis (MMFDFA) [34]

### Generalized Fractal Dimensions and Legendre Spectrum

In practice, in order to quantify local densities of a considered set (an image), the mass probability in the *i*th box is estimated as [35]:

5 $${P}_{i}\left(L\right)= {N}_{i}(L)/{N}_{T}$$

and varies as:

6 $${P}_{i}\left(L\right)\sim {L}^{{\alpha }_{i}}$$

where ${N}_{i}(L)$ is the number of pixels in the *i*th box, ${N}_{T}$ is the total mass of the set, and ${\alpha }_{i}$ is the Hölder exponent that reflect the local behavior of the mass probability ${P}_{i}(L)$ around the center of each box of size $L$, it can be estimated as [35]:

7 $${\alpha }_{i}= ln {P}_{i}(L)/ln(L)$$

The number of boxes $N(\alpha )$ where the probability ${P}_{i}$ has similar exponent values between $\alpha$ and $\alpha +\Delta \alpha$ follows the power law with the box size $L$ and the multifractal spectrum $f(\alpha )$ [35]:

8 $$N(\alpha )\sim {L}^{-f(\alpha )}$$

Since multifractals are affected by distortions, the process of multifractal analysis is equivalent to applying warp filters to an image to analyze imperceptible features. Warp filters are a set of arbitrary exponents traditionally denoted by the symbol “$q$” usually manipulated from a set bracketing 0 in a symmetrical way (e.g., from − 5 to 5). Therefore, a characterization of multifractal measures can be made through the scaling of the *q*th moments of ${P}_{i} (L)$ distributions in the form:

9 $$\sum_{i=1}^{N\left(L\right)}{P}_{i}^{q}\left(L\right)={L}^{\tau \left(q\right)}={L}^{(q-1){D}_{q}}$$

The exponent $\tau (q)$ is called the mass exponent of the *q*th order moment, and ${D}_{q}$, *q* *€* *R* are the generalized fractal dimensions defined from Eq. [9] as:

10 $${D}_{q}=\underset{L\to 0}{\mathit{lim}}\frac{1}{q-1}\frac{\mathit{log}\sum_{i=1}^{N(L)}{P}_{i}^{q}(L)}{logL}$$

where $\sum_{i=1}^{N(L)}{P}_{i}^{q}(L)$ is the mean of the distribution of the distorted probability mass for a size $L$, and the generalized dimension ($Dq$) is determined for each $q$ where each mass is distorted by being raised to $q$. So, $q$ can be considered as a “microscope” allowed to explore different regions of the ${P}_{i}$ distribution. Low values of $q$ favor boxes with low ${P}_{i}(L)$ (low irregularities) and high values of $q$ favor boxes with high values of ${P}_{i}(L)$ (high irregularities). In other words, for $q > 1$, ${D}_{q}$ represents the more singular regions, and for $q < 1$, it accentuates the less singular regions [36].

The relation between the power exponents $f(\alpha )$ and the exponent $\tau (q)$ is established via the Legendre transformation:

11 $$f\left[\alpha \left(q\right)\right]=q\alpha \left(q\right)-\tau (q)$$

and

12 $$\alpha \left(q\right)=\frac{d\tau (q)}{dq}$$

However, the determination of $f(\alpha )$ needs to smooth the ${D}_{q}$ curve and then use the Legendre transformation. The smoothing operation provides errors in the estimation of $f(\alpha )$ and misses phase transitions when it exhibits discontinuities. In order to avoid these numerical errors, Chhabra and Jensen proposed a method for a direct calculation of the multifractal spectrum using the following formulas [28]:

First, a family of normalized measures ${\mu }_{i}(q,L)$ was constructed where the probabilities in the boxes of size $L$ are:

13 $${\mu }_{i}\left(q,L\right)=\frac{{P}_{i}^{q}(L)}{\sum_{i=1}^{N(L)}{P}_{i}^{q}(L)}$$

Then, the direct computation of $f(q)$ and $\alpha (q)$ values is:

14 $$\alpha \left(q\right)=\underset{L\to 0}{\mathit{lim}}\frac{\sum_{i=1}^{N(L)}{\mu }_{i}\left(q,L\right)log[{P}_{i}\left(L\right)]}{logL}$$

15 $$f\left(q\right)=\underset{L\to 0}{\mathit{lim}}\frac{\sum_{i=1}^{N(L)}{\mu }_{i}\left(q,L\right)log[{\mu }_{i}\left(q,L\right)]}{logL}$$

For each $q$, values of $\alpha (q)$ and $f(q)$ are obtained from the slope of plots of the numerators of Eqs. (14) and (15) vs. $log L$ over the entire range of $L$ values considered. The $f(q)$ and $\alpha (q)$ functions obtained over a given $\Delta q$ were used to construct the $f(\alpha )$-spectrum as an implicit function of $q$ and $L$.

### Multifractal Detrended Moving Average (MFDMA)

The two-dimensional multifractal detrended moving average (2D-MFDMA) algorithm is described as follows [33]: Consider a surface of possible multifractal properties which can be denoted by a two-dimensional matrix $X({i}_{1},{i}_{2})$*,* with ${i}_{1}=\mathrm{1,2},...,{N}_{1}$, and ${i}_{2}=\mathrm{1,2},...,{N}_{2}$.

Step 1: Calculate the sum $Y({i}_{1},{i}_{2})$

The first step consist to calculate the sum $Y({i}_{1},{i}_{2})$ in a sliding window with size ${n}_{1} \times {n}_{2}$, where ${n}_{1}\le {i}_{1}\le {N}_{1}-\left({(n}_{1}-1\right){\theta }_{1}$ and ${n}_{2}\le {i}_{2}\le {N}_{2}-\left({(n}_{1}-1\right){\theta }_{2}$. *θ*_*1*_ and *θ*_*2*_ are two position parameters that vary in the range $[0 \space\ 1]$. Specifically, we extract a sub-matrix $Z=X({u}_{1},{u}_{2})$ with size ${n}_{1}\times {n}_{2}$ from the matrix $X$, where ${i}_{1}-{n}_{1}+1 \le {u}_{1}\le {i}_{1}$ and ${i}_{2}-{n}_{2}+1 \le {u}_{2}\le {i}_{2}$. We can calculate the sum $Y({i}_{1},{i}_{2})$ of $Z$ as follows:

16 $$Y\left({i}_{1},{i}_{2}\right)=\sum_{{j}_{1}=1}^{{n}_{1}}\sum_{{j}_{2=1}}^{{n}_{2}}Z({j}_{1},{j}_{2})$$

Step 2: Determine the moving average function $\tilde{Y }\left({i}_{1,}{i}_{2}\right)$.

First, we extract a sub-matrix $$W=X({u}_{1},{u}_{2})$$ with size $${n}_{1} \times {n}_{2}$$ from the matrix $$X$$, where $${k}_{1}-\lceil\left({n}_{1}-1\right)(1-{\theta }_{1})\rceil$$$$\le {u}_{1}\le {k}_{1}-\left({n}_{1}-1\right){ \theta }_{1}$$ and $${k}_{2}-\lceil\left({n}_{2}-1\right)(1-{\theta }_{2})\rceil\le {u}_{2}$$$$\le {k}_{2}-\left({n}_{2}-1\right){ \theta }_{2}$$, where $$\lceil\left({n}_{1}-1\right)(1-{\theta }_{1})\rceil+1\le {k}_{1}\le$$$${N}_{1}-\left({n}_{1}-1\right){ \theta }_{1}$$ and $$\lceil\left({n}_{2}-1\right)(1-{\theta }_{2})\rceil+1\le {k}_{2}\le {N}_{2}-$$$$\left({n}_{2}-1\right){ \theta }_{2}$$. Then, we calculate the cumulative sum of the matrix $$\tilde{W }({m}_{1},{m}_{2})$$ of *W*:

17 $$\tilde{W }\left({m}_{1},{m}_{2}\right)=\sum_{{d}_{1}=1}^{{m}_{1}}\sum_{{d}_{2=1}}^{{m}_{2}}W({d}_{1},{d}_{2})$$

where $$1\le {m}_{1}\le {n}_{1}$$ and $$1\le {m}_{2}\le {n}_{2}$$.

The moving average function $\tilde{Y }\left({i}_{1,}{i}_{2}\right)$ can be calculated as follows:

18 $$\tilde{Y }\left({i}_{1,}{i}_{2}\right)=\frac{1}{{n}_{1}{n}_{2}}\sum_{{m}_{1}=1}^{{n}_{1}}\sum_{{m}_{2=1}}^{{n}_{2}}\tilde{W }\left({m}_{1},{m}_{2}\right)$$

Step 3: The residual matrix $\varepsilon \left({i}_{1},{i}_{2}\right)$

The matrix is detrended by removing the moving average function $\tilde{Y }\left({i}_{1,}{i}_{2}\right)$ from $Y\left({i}_{1},{i}_{2}\right)$ to obtain the residual matrix $\varepsilon \left({i}_{1},{i}_{2}\right)$:

19 $$\varepsilon \left({i}_{1},{i}_{2}\right)=Y\left({i}_{1},{i}_{2}\right)-\tilde{Y }\left({i}_{1},{i}_{2}\right)$$

Step 4: The detrended fluctuation function.

The residual matrix $\varepsilon \left({i}_{1},{i}_{2}\right)$ is partitioned into ${N}_{{n}_{1}}\times {N}_{{n}_{1}}$ disjoint rectangle segment of the same size. Each segment can be denoted by ${\varepsilon }_{{v}_{1},{v}_{2}}$ such that ${\varepsilon }_{{v}_{1},{v}_{2}}\left({i}_{1},{i}_{2}\right)=\varepsilon \left({I}_{1}+{i}_{1},{I}_{2}+{i}_{2}\right)$ for $1\le {i}_{1}\le {n}_{1}$ and $1\le {i}_{2}\le {n}_{2}$, where ${I}_{1}=({v}_{1}-1){n}_{1}$ and ${I}_{2}=({v}_{2}-1){n}_{2}$. The detrended fluctuation ${F}_{{v}_{1},{v}_{2}}\left({n}_{1},{n}_{2}\right)$ of segment ${\varepsilon }_{{v}_{1},{v}_{2}}\left({i}_{1},{i}_{2}\right)$ can be calculated as follow:

20 $${F}_{{v}_{1{v}_{2}}}^{2}\left({n}_{1},{n}_{2}\right)=\frac{1}{{n}_{1}{n}_{2}}\sum_{{i}_{1}=1}^{{n}_{1}}\sum_{{i}_{2}=1}^{{n}_{2}}{\varepsilon }_{{v}_{1}{v}_{2}}^{2}({i}_{1},{i}_{2})$$

Step 5: The *q*th order overall fluctuation function ${f}_{q}(n)$ is calculated as follows:

21 $${F}_{q}\left(n\right)={\left\{\frac{1}{{N}_{{n}_{1}}{N}_{{n}_{2}}}\sum_{{v}_{1}=1}^{{N}_{{n}_{1}}}\sum_{{v}_{2}=1}^{{N}_{{n}_{2}}}{F}_{{v}_{1},{v}_{2}}^{q}({n}_{1},{n}_{2})\right\}}^\frac{1}{q}$$

${F}_{q}\left(n\right)$ is a vector obtained from the detrended fluctuation of each segment for each value of $q$. Then, it is used to calculate the multifractal scaling exponent $\tau (q)$, ${n}^{2}={(n}_{1}^{2}+{n}_{2}^{2})/2$ and $q$, mathematically, can take any real values except $q=0$. In practice, if $q=0$, ${F}_{q}\left(n\right)$ is given by: ${F}_{q}\left(n\right)={exp}^{\frac{1}{2} \frac{1}{{N}_{{n}_{1}}{N}_{{n}_{2}}}\sum_{{v}_{1}=1}^{{N}_{{n}_{1}}}\sum_{{v}_{2}=1}^{{N}_{{n}_{2}}}\mathit{log}{F}_{{v}_{1},{v}_{2}}^{2}({n}_{1},{n}_{2})}$

Step 6: Varying the segment sizes ${n}_{1}$ and ${n}_{2}$, we can determine the power-law relation between the *q-*order overall fluctuation function ${F}_{q}\left(n\right)$ and the scale $n$:

22 $${F}_{q}\left(n\right)\sim {n}^{H(q)}$$

For each *q*, we can get the corresponding traditional $\tau \left(q\right)$ function through:

23 $$\tau \left(q\right)=qH\left(q\right)-{D}_{f}$$

and obtain the singularity strength function $\alpha (q)$ and the multifractal spectrum $f(\alpha )$ via Legendre transform.

### Comparison Between the Two Methods

The generalized fractal dimensions method is the most used in literature due to its simplicity of its implementation and robustness. Compared to MFDMA, the computation time is short and fast (Table 3) and only two parameters need to be adjusted (Table 4):

- Box sizes  $L$: are powers of two, i.e.,  $L = 1, 2, 4 ... {2}^{p}$, where  $P$ is the smallest integer such that  $max(size(Image)) \le {2}^{p}$.
- *q*-orders: described previously. In this paper, we took  $q=[-4:0.1:4]$*.*

**Table 3:** Computation time of each method according to the image size

Image (ROI) size	Generalized fractal dimension	MFDMA
64 × 64	2.243523 s	2.767289 s
256 × 256	2.225408 s	17.780042 s

**Table 4:** Parameters to be set for each method

Generalized fractal dimension	MFDMA
- Box size - q-orders	- Moving average detrending - Sample size—Scaling range - q-orders

## Database

**Fig. 1:**
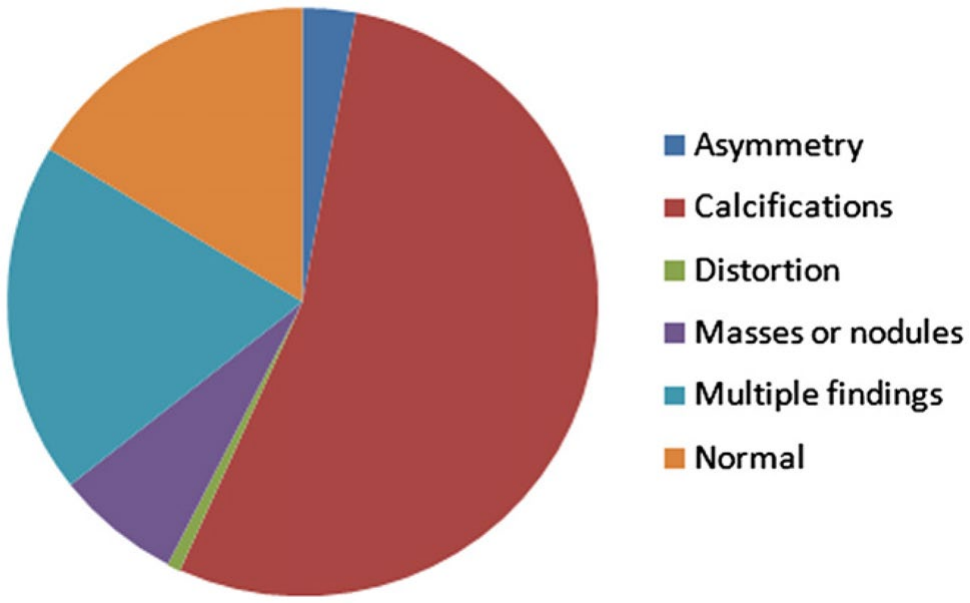
Chart describing findings in the INbreast database [37]

**Fig. 2:**
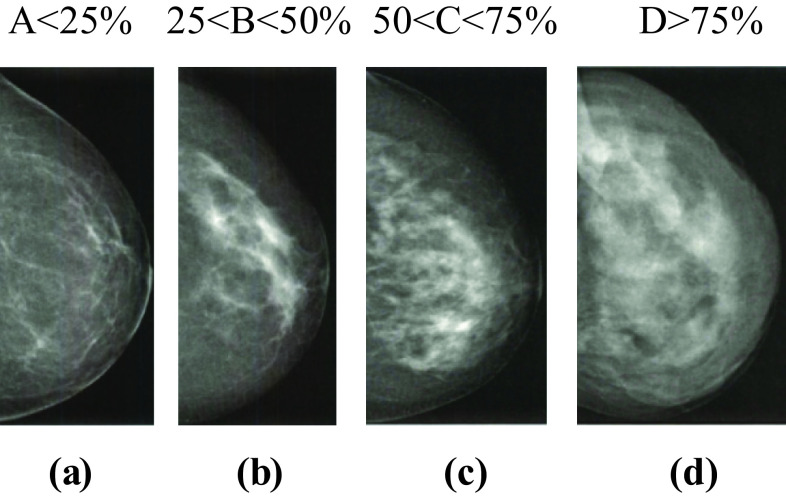
BI-RADS classification of the breast density. **a** Almost entirely fat tissue, **b** scattered fibro-glandular tissue, **c** heterogeneously dense tissue, and **d** extremely dense tissue

There are several mammographic image databases which are used by researchers in the breast analysis field. In this paper, we used the INbreast database. This database was acquired at the Breast Center of Porto. The image matrices are 3328 × 4084 or 2560 × 3328 pixels saved in DICOM format. It has FFDM (full-field digital mammograms) images from screening, diagnostic, and follow-up cases with a total of 115 cases (a total of 410 images) of which 90 cases (MLO and CC) are from women with both breasts affected (four images per case) and 25 cases are from mastectomy patients (two images per case). It includes several types of lesions: masses, calcifications, asymmetries, architectural distortions, and multiple findings (Fig. 1) and provides information regarding patient’s age at the time of image acquisition, family history, BI-RADS classification of the breast density (Fig. 2), and abnormality [37].

**Fig. 3:**
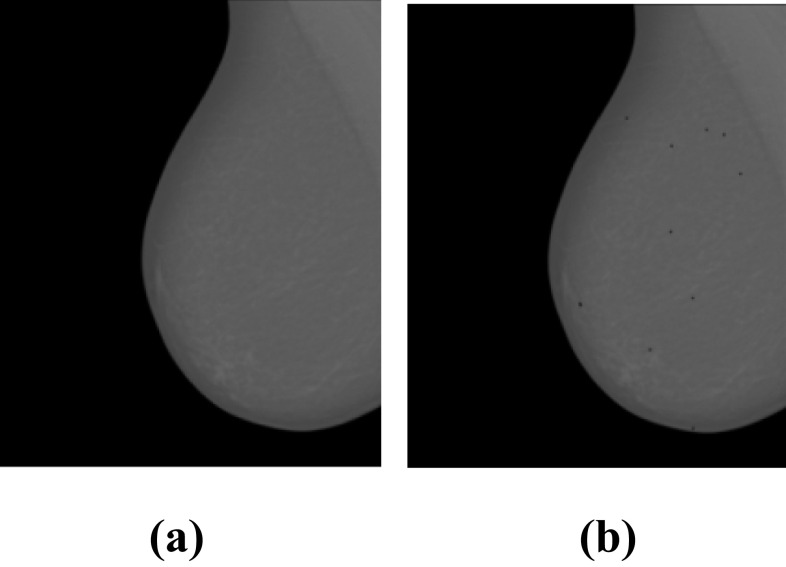
Example of mammogram containing MCs from INbreast database. **a** Original mammogram and **b** original mammogram with MCs’s annotations

The database contains 308 images of calcifications of different BI-RADS breast density classes (Fig. 2, Table 5). Thus, 6880 calcifications were individually identified in 299 images. Annotations were made by a specialist in the field, and validated by a second specialist (Fig. 3). These specialists are experts in reading mammograms. A detailed contour of the finding was made. An ellipse enclosing the entire cluster was adopted to annotate the clusters of MCs [37].

**Table 5:** Number of mammograms containing MCs of each BI-RADS class

BI-RADS class	A	B	C	D
No. of MCs cases	102	105	81	20

## Proposed Method

**Fig. 4:**
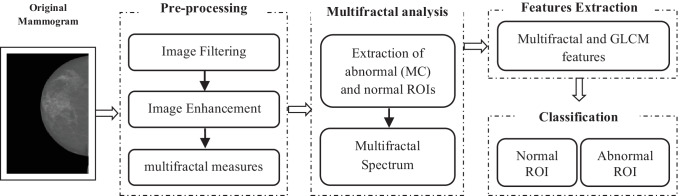
Global diagram of the proposed approach

In this paper, we propose an approach based on multifractal analysis for the characterization and classification of normal and abnormal ROIs to detect MCs. Figure 4 shows the global diagram of the proposed approach. Our method is divided into five steps: pre-processing, ROI extraction, multifractal analysis, feature extraction, and classification.

### Pre-Processing

#### Image Filtering

Images of the INbreast database are of high quality and do not contain labels or artifacts, so they do not require a filtering step. But they are of large size and contain many empty rows and columns which represent the background of the mammogram (represented in black). So, in order to reduce the computation time of the next steps, the mammogram is filtered from this black background by removing the empty rows and columns and keeping only the breast [25].

#### Image Enhancement

Considering breast density as haze in mammography, we use a low-light image enhancement algorithm based on the haze removal technique [38] to reduce breast density and highlight MCs in relation to density [25].

#### α-image (Multifractal Measures)

α-image is constructed from the multifractal measures and the estimated Hölder exponents ${\alpha }_{p}$. For estimating Hölder exponents ${\alpha }_{p}$, natural logarithms of measure value $\mathrm{ln}({\mu }_{p}\left(m,n\right))$ and of the window size $\mathrm{ln}(L)$ (Eq. 2) are calculated and plotted corresponding points in bi-logarithmic diagram $\mathrm{ln}({\mu }_{p}\left(m,n\right))$ vs. $\mathrm{ln}(L)$ where 03 boxes of 1 × 1, 3 × 3, and 5 × 5 pixels in size were considered. Then, the limiting value of $\alpha (m,n)$ is estimated as the slope of the linear regression line. All steps are detailed in the previous work [25]. The final result represents the enhanced mammogram.

### ROI Extraction and Multifractal Analysis

Normal and abnormal ROIs containing MCs are extracted from the enhanced image. Based on the expert’s annotations, the coordinates of MCs are extracted, and then the ROI is cropped such that these coordinates represent the center of the ROI. For a normal ROI, the coordinates of the center are chosen randomly where there is no annotation. The ROIs are nonoverlapping of size 64 × 64 pixels. Then, for each extracted ROI, the multifractal spectrum is calculated using the two different methods described in the previous section.

### Feature Extraction

#### Multifractal Features

**Fig. 5:**
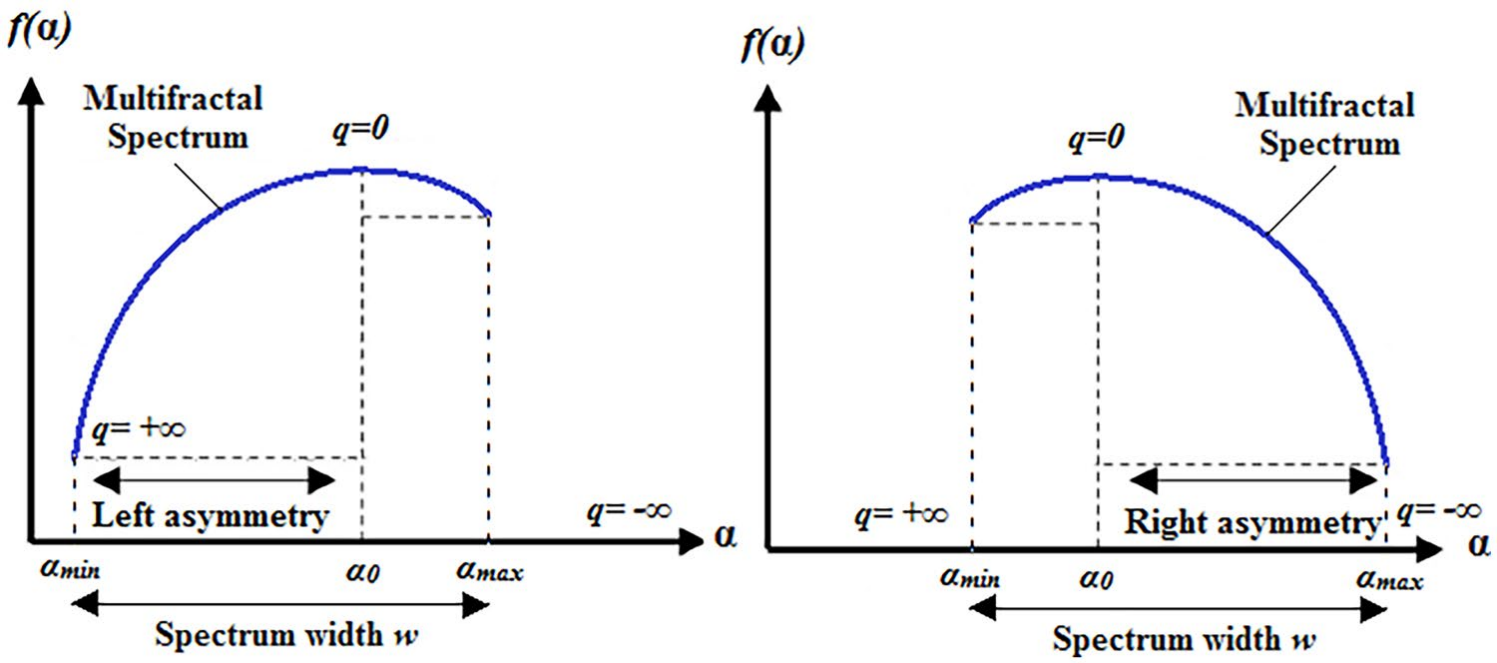
Schematic representation of the multifractal spectrum and the main parameters

From each multifractal spectrum of each ROI, five multifractal parameters are extracted: Hölder exponent ${\alpha }_{0}$ (correspending to the maximum of the spectrum), spectrum width ($w={\alpha }_{max}- {\alpha }_{min}$), $R= {\alpha }_{0}- {\alpha }_{min}$, $L= {\alpha }_{max}- {\alpha }_{0}$, and Asymmetry ($A=({\alpha }_{0}- {\alpha }_{min})/( {\alpha }_{max}- {\alpha }_{0})$) (Fig. 5).

The Hölder exponent ${\alpha }_{0}$ measures the intensity of the local irregularities present in the image [39]. The parameter *Δα* is a measure determining the degree of multifractality. The smaller *Δα* indicates that the function tends to be monofractal and the larger one indicates the enhancement of multifractality [40]. Asymmetry $A$ measures the symmetry of the spectrum. It is null for symmetrical shapes, positive or negative for left and right asymmetric shapes, respectively [41]. An asymmetric spectrum on the right corresponds to a concentration of irregularities in large structures and conversely, an asymmetrical spectrum on the left corresponds to a concentration of irregularities in fine structures [42].

#### Gray Level Co-occurrence Matrix (GLCM)

GLCM is defined as the distribution of co-occurring values at a given offset. It consists of identifying patterns of pairs of pixels separated by a distance $d$ in a direction $\theta$ by calculating how often a pixel with a gray-level value $i$ occurs either horizontally ($0^\circ$), vertically ($90^\circ$) or diagonally ($-45^\circ$ or $-135^\circ$) to adjacent pixels with the value $i$.

In this paper, since MCs are small structures of different sizes and shape, we extracted GLCM metrics for single pixel distances (an offset $d=1$) along the horizontal direction ($\theta =0^\circ$). If we apply large distances or different directions, the GLCM can miss detailed information. The chosen metrics also allow us to minimize execution time.

After the creation of the GLCM, several statistics can be derived providing information about the texture of the image [43]:

$$\mathrm{Contrast}= \sum_{i,j}{\left|i-j\right|}^{2} P\left(i,j\right), \mathrm{Correlation}= \sum_{i,j}\frac{\left(i-\mu i\right)\left(j-\mu j\right)}{{\sigma }_{i}{\sigma }_{j}}P\left(i,j\right)$$

$$\mathrm{Energy}=\sum_{i,j}{P(i,j)}^{2}, \mathrm{Homogeneity}= \sum_{i,j}\frac{1}{1+{\left(i-j\right)}^{2}}P(i,j)$$

where

$${\varvec{P}}\left({\varvec{i}},{\varvec{j}}\right)=\mathrm{the\ probability\ density\ function\ of\ gray}-\mathrm{level\ pairs}$$

$${\mu}_{i}=\sum_{i}i\sum_{j}P\left(i,j\right), {\mu}_{j}=\sum_{j}j\sum_{i}P(i,j), {\sigma }_{i}=\sum_{i}{(i-{\mu}_{i})}^{2}\sum_{j}P\left(i,j\right), {\sigma }_{j}=\sum_{j}{(j-{\mu }_{j})}^{2}\sum_{i}P\left(i,j\right)$$

### Classification

Classification based on machine learning techniques is a two-step process: the learning step where the model is developed from given training data and the prediction step where the trained model is used to predict the response of given data. Generally, it is a supervised machine-learning that use training data and associated labels during the model learning process. The objective is to predict output labels of input data related to what the model has learned during the training phase. Therefore, each output response belongs to a specific class. Many supervised machine-learning algorithms have been proposed in the literature [44]. The most popular are support vector machines (SVM), K-nearest neighbors (KNN), and decision trees (DT) which are used in this research to classify ROIs in which the output is either normal (healthy) or abnormal (MCs) case.

#### Support Vector Machine (SVM)

Support vector machines (SVM) are one of the main supervised machine-learning algorithms that are not only accurate but also highly robust. In the SVM algorithm, each data item is plotted as a point in *n*-dimensional space (where *n* is number of features) with the value of each feature being the value of a particular coordinate. The objective is to find the most appropriate classification function by making a comparison of the separating hyperplane that goes through the center of the two classes, separating the two. The role of SVM is to increase the margin (the shortest possible space between the hyperplane point and the closely located data points) to the maximum between the two classes [44, 45].

#### Decision Tree (DT)

A decision tree is a hierarchical supervised learning model. The goal of using a DT is to create a training model that can be used to predict the class of the target variable by learning simple decision rules **(**in the form of yes or no questions) inferred from prior data (training data). A DT is made up of internal decision nodes and terminal leaves. Each decision node uses a test function that labels the branches with discrete scores. A test is used at every node with an input, and one of the branches is chosen depending on the result. This process starts from the root with the complete training information, the best split must be checked in each phase. It divides the training data into two or more classes. Then, we continue to divide recursively with the relevant subset until there is no longer any need to split; at this stage, a leaf node is generated and labeled [44].

#### K-Nearest Neighbors (KNN)

In k-nearest neighbors classification, examples are classified based on the class of their nearest neighbors (more than one neighbor). The K-NN classification has two stages: the first is the determination of the nearest neighbors, and the second is the determination of the class using those neighbors. When classification needs to be determined for an unlabeled object, the distance metric between the labeled object and the unlabeled object is calculated. The k-nearest neighbors are selected based on this distance metric. Thereby, the identification of the k-nearest neighbors is attained. Therefore, the nearest neighbors’ class labels are employed in order to identify the object’s class label [44, 46].

To assess the performance of our approach, the extracted features are fed to the classifiers and classification accuracy, sensitivity, and specificity are calculated. The SVM classifier was trained using standardized predictors and the second-order polynomial kernel. The KNN classifier was trained using standardized Euclidean distance metric. The DT classifier was trained without using specific options.

In order to split our database into training and testing sets, the k-fold cross validation method is used. It consists to split the database into k groups, for each k, the group is taken as the test data and the remaining groups are taken as training data. This process (cross validation process) is repeated k times. In this paper, each classifier was tested using 5-folds cross validation.

Let TP, FP, TN, and FN be the number of true positives, false positives, true negative, and false negatives, respectively, and sensitivity, specificity, and accuracy are defined by:

$$\mathrm{Sensitivity}=\frac{TP}{TP+FN}\times 100\%$$

$$\mathrm{Specificity}=\frac{TN}{TN+FP}\times 100\mathrm{\%}$$

$$\mathrm{Accuracy}=\frac{TP+TN}{TP+TN+FP+FN}\times 100\mathrm{\%}$$

## Results and Discussion

The proposed system was tested and validated using MATLAB R2019b, on a personal computer with an Intel (R) Core (TM) i5-3230 M CPU processor and 8 Go RAM, running under Windows 7 operating system.

**Fig. 6:**
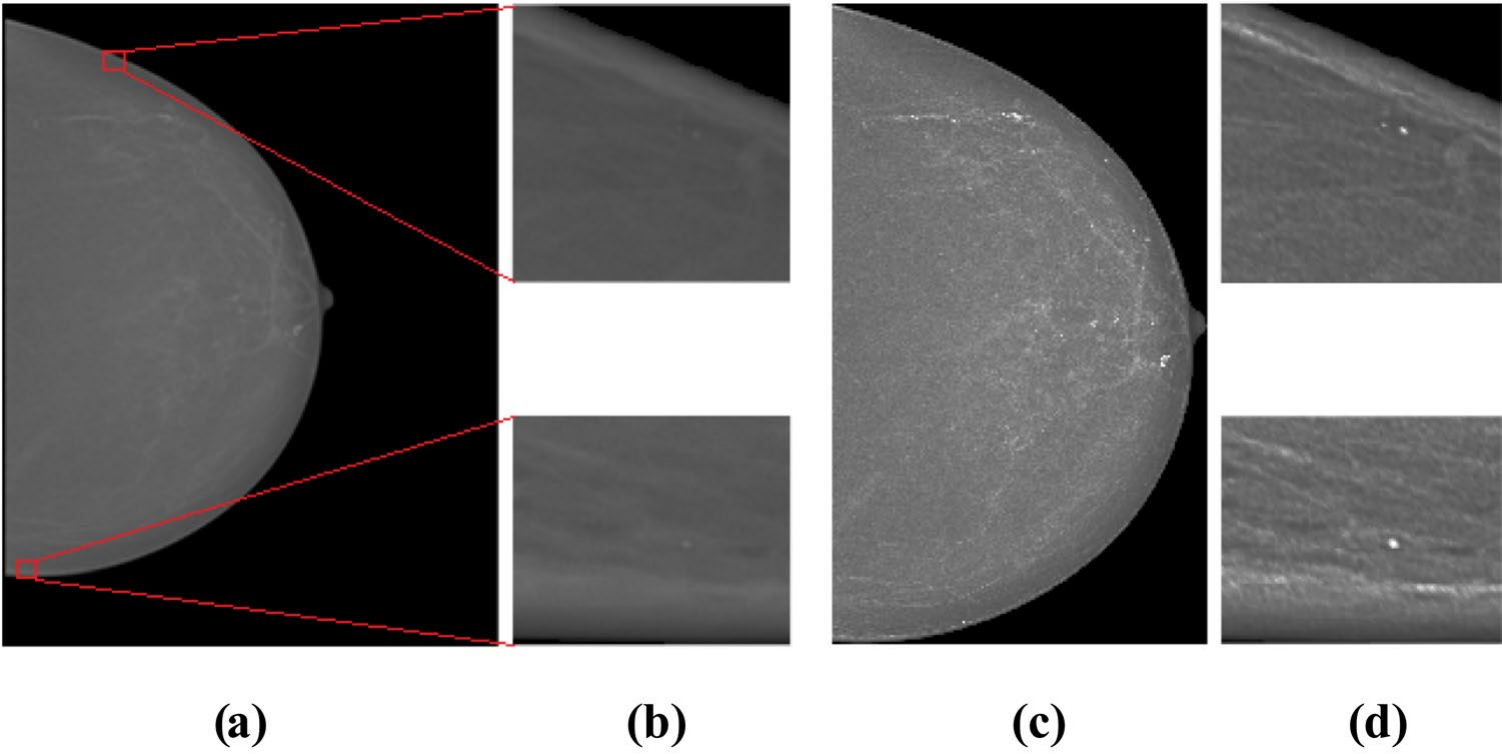
Result of mammogram pre-processing using proposed approach, case “20,587,320” of INbreast database. **a** Original mammogram. **b** Original ROIs. **c** Enhanced mammogram. **d** Enhanced ROIs with highlighted MCs

**Fig. 7:**
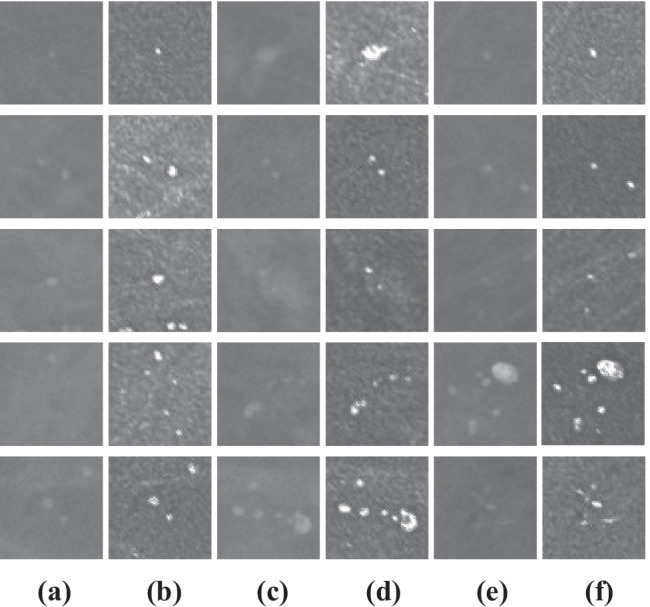
Examples of abnormal ROIs containing MCs. **a**, **c**, **e** Original ROI. **b**, **d**, **f** Pre-processed ROI

**Fig. 8:**
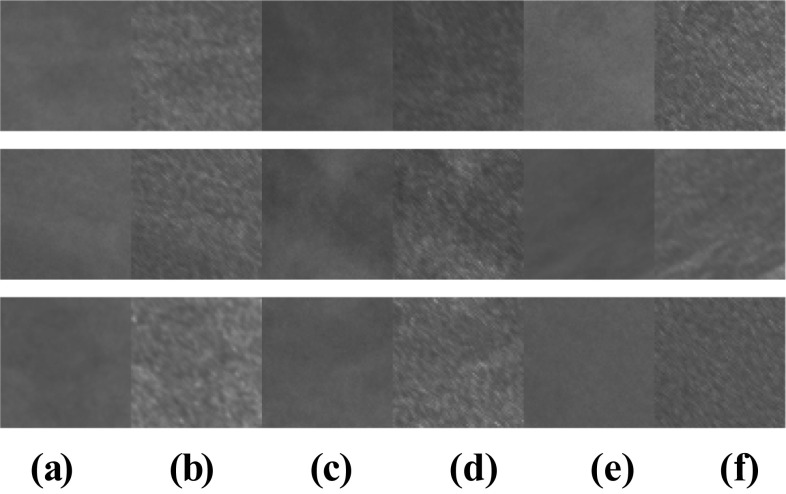
Examples of normal ROIs. **a**, **c**, **e** Original ROI. **b**, **d**, **f** Pre-processed ROI

After the pre-processing step (Fig. 6), normal ROIs and abnormal ROIs (containing MCs) of size 64 × 64 pixels were extracted from the pre-processed mammograms based on the expert’s annotations. The size of the ROI influences the multifractal spectrum and its parameters. If the ROI’s size is larger, the ROI contains more singularities (structures); therefore, it can negatively impact the classification. If the ROI’s size is smaller, the classification results will be better. Also, the choice of this size allows us to reduce the computation time of the multifractal spectrum. Figures 7 and 8 compare some examples of ROIs obtained before (without pre-processing step) and after pre-processing (with pre-processing step). The influence of pre-processing was explained, evaluated, and discussed in the previous work [25].

**Fig. 9:**
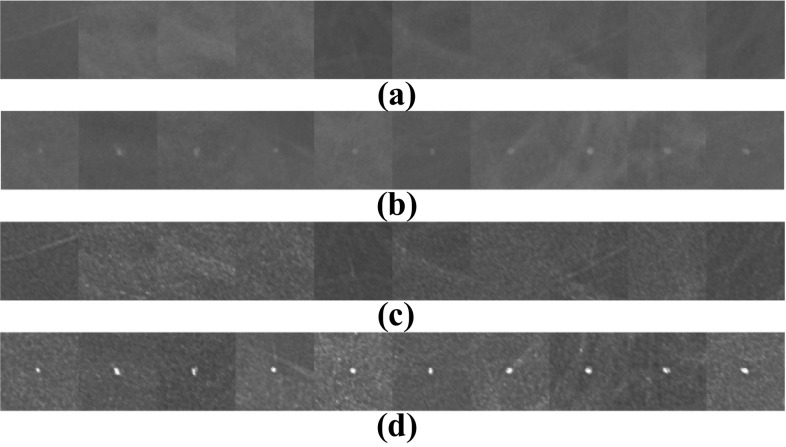
Examples of normal and abnormal ROIs. **a** 10 Original normal ROIs. **b** 10 original abnormal ROIs. **c**, **d** The results of their pre-processing respectively

**Fig. 10:**
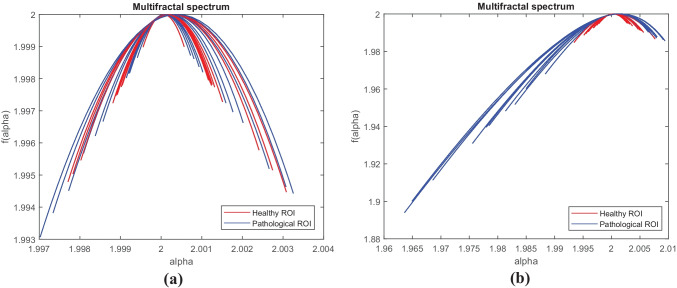
Multifractal spectrums of normal (red) and abnormal ROIs (blue). **a** Before pre-processing. **b** After pre-processing

The next step is to compute the multifractal spectrum of each ROI (normal and abnormal) and extract the parameters described in the previous section. To illustrate this, we took 10 examples of normal ROI and 10 examples of abnormal ROI (Fig. 9), we calculated the multifractal spectrum using the generalized fractal dimensions method of each ROI before and after pre-processing, then, the multifractal parameters are extracted. Figure 10 shows the result of obtained multifractal spectrums of ROIs before and after pre-processing. The extracted parameters are presented in Tables 6 and 7.

**Table 6:** Extracted parameters from the multifractal spectrum of normal ROI. (a) Before pre-processing, (b) after pre-processing

	*α*_*0*_		*w*		*R*		*L*		*A*	
	(a)	(b)	(a)	(b)	(a)	(b)	(a)	(b)	(a)	(b)
im_01	2.0001	2.0004	0.0023	0.0063	0.0012	0.0035	0.0011	0.0028	1.1075	1.2393
im_02	2.0002	2.0007	0.0026	0.0105	0.0013	0.0055	0.0013	0.0050	0.9485	1.1017
im_03	2.0003	2.0009	0.0054	0.0143	0.0026	0.0075	0.0027	0.0068	0.9565	1.1049
im_04	2.0001	2.0006	0.0022	0.0088	0.0011	0.0046	0.0011	0.0041	1.0424	1.1163
im_05	2.0001	2.0003	0.0022	0.0049	0.0011	0.0025	0.0011	0.0024	0.9804	1.0311
im_06	2.0003	2.0006	0.0049	0.0095	0.0025	0.0051	0.0024	0.0044	1.0183	1.1493
im_07	2.0001	2.0003	0.0010	0.0049	0.0005	0.0025	0.0005	0.0024	1.0072	1.0618
im_08	2.0003	2.0006	0.0044	0.0101	0.0022	0.0055	0.0021	0.0046	1.0561	1.1882
im_09	2.0002	2.0004	0.0025	0.0069	0.0013	0.0037	0.0011	0.0032	1.1614	1.1247
im_10	2.0001	2.0003	0.0021	0.0055	0.0011	0.0029	0.0010	0.0026	1.0540	1.1312

**Table 7:** Extracted parameters from multifractal spectrum of abnormal ROI. (a) Before pre-processing, (b) after pre-processing

	*α*_*0*_		*w*		*R*		*L*		*A*	
	(a)	(b)	(a)	(b)	(a)	(b)	(a)	(b)	(a)	(b)
im_01	2.0001	2.0007	0.0013	0.0168	0.0007	0.0124	0.0006	0.0045	1.0404	2.7652
im_02	2.0003	2.0013	0.0043	0.0431	0.0025	0.0377	0.0018	0.0054	1.4420	6.9454
im_03	2.0002	2.0011	0.0030	0.0281	0.0016	0.0227	0.0014	0.0054	1.1525	4.1829
im_04	2.0003	2.0011	0.0046	0.0259	0.0022	0.0197	0.0024	0.0062	0.9241	3.1956
im_05	2.0001	2.0011	0.0019	0.0229	0.0010	0.0161	0.0009	0.0068	1.0744	2.3755
im_06	2.0001	2.0008	0.0017	0.0216	0.0009	0.0178	0.0008	0.0038	1.0958	4.6315
im_07	2.0002	2.0013	0.0034	0.0300	0.0018	0.0233	0.0016	0.0067	1.1788	3.4643
im_08	2.0004	2.0016	0.0057	0.0445	0.0030	0.0366	0.0027	0.0079	1.1109	4.6651
im_09	2.0004	2.0015	0.0062	0.0405	0.0034	0.0329	0.0029	0.0076	1.1778	4.3309
im_10	2.0001	2.0011	0.0016	0.0309	0.0009	0.0256	0.0007	0.0054	1.2305	4.7778

From the figures and tables above, we notice that before pre-processing, the normal ROIs were confused with the abnormal ROIs containing only one MC, and the multifractal spectrum could not differentiate between them. But after pre-processing, a very good discrimination of these ROIs was obtained from the multifractal spectrum and the extracted parameters.

According to Table 8, before pre-processing, we notice that the variation intervals of each parameter are very close to the normal and abnormal ROIs. After pre-processing, a large difference in these intervals is recorded, hence a better characterization and discrimination between the normal and the abnormal ROIs.

**Table 8:** Interval of variation of each multifractal parameter before and after pre-processing

	Before pre-processing		After pre-processing	
	Normal ROIs	Abnormal ROIs	Normal ROIs	Abnormal ROIs
*α*_*0*_	[2.0001–2.0003]	[2.0001–2.0004]	[2.0003–2.0009]	[2.0007–2.0016]
*w*	[0.001–0.0054]	[0.0013–0.0062]	[0.0049–0.0143]	[0.0168–0.0445]
*R*	[0.0005–0.0026]	[0.0007–0.0034]	[0.0025–0.0075]	[0.0124–0.0377]
*L*	[0.0005–0.0027]	[0.0006–0.0029]	[0.0024–0.0068]	[0.0038–0.0079]
*A*	[0.9485–1.1614]	[0.9241–1.442]	[1.0311–1.2393]	[2.3755–6.9454]

## Results of ROI Classification

The proposed approach was applied to each BI-RADS class of the INbreast database (Table 9). After extraction of normal and abnormal ROIs where only ROIs with individual MC were considered, the multifractal spectrum was calculated using the two methods described in the previous section. Extracted parameters are used for classification of ROIs using the three classifiers. Obtained results are mentioned in Tables 10, 11 and 12. Table 13 presents the comparison of our results with the state of the art.

**Table 9:** ROI’s number of each class

BI-RADS Class		A	B	C	D
ROIs number	Normal	300	376	368	300
	Abnormal (MC)	300	376	368	300
	Total	600	752	736	600

**Table 10:** Classification of normal and abnormal ROIs of each BI-RADS class using the generalized fractal dimension method

	BI-RADS A					
	Before pre-processing			After pre-processing		
Classifier	Sensitivity	Specificity	Accuracy	Sensitivity	Specificity	Accuracy
KNN	83.55%	82.66	83.17%	100%	99.67%	99.83%
SVM	91.06%	76.35%	83.83%	100%	99.64%	99.83%
DT	80.01%	79.36%	79.83%	99.68%	99.66%	99.67%
	BI-RADS B					
	Before pre-processing			After pre-processing		
Classifier	Sensitivity	Specificity	Accuracy	Sensitivity	Specificity	Accuracy
KNN	81.14%	78.59%	79.79%	99.49%	100%	99.74%
SVM	92.82%	76.95%	84.84%	99.75%	99.15%	99.47%
DT	81.35%	79.79%	80.59%	99.78%	100%	99.87%
	BI-RADS C					
	Before pre-processing			After pre-processing		
Classifier	Sensitivity	Specificity	Accuracy	Sensitivity	Specificity	Accuracy
KNN	66.21%	71.62%	68.89%	97.27%	98.02%	97.69%
SVM	97.81%	53.27%	75.54%	98.12%	96.51%	97.28%
DT	73.93%	71.85%	72.83%	96.73%	96.41%	96.60%
	BI-RADS D					
	Before pre-processing			After pre-processing		
Classifier	Sensitivity	Specificity	Accuracy	Sensitivity	Specificity	Accuracy
KNN	50.14%	53.68%	51.83%	90%	91.32%	90.67%
SVM	30.21%	77.15%	52.83%	94.01%	92.95%	93.50%
DT	55.04%	52.65%	54%	87.48%	87.87%	87.83%

**Table 11:** Classification of normal and abnormal ROIs of each BI-RADS class using the MFDMA method

	BI-RADS A					
	Before pre-processing			After pre-processing		
Classifier	Sensitivity	Specificity	Accuracy	Sensitivity	Specificity	Accuracy
KNN	75.76%	74.46%	75.17%	96.63%	97.29%	97%
SVM	96.09%	57.40%	76.83%	97.31%	91.88%	94.67%
DT	75.18%	71.28%	73.17%	96.33%	95.99%	96.17%
	BI-RADS B					
	Before pre-processing			After pre-processing		
Classifier	Sensitivity	Specificity	Accuracy	Sensitivity	Specificity	Accuracy
KNN	81%	78.88%	79.92%	96.53%	95.44%	96.01%
SVM	96.66%	68.74%	82.71%	97.87%	94.36%	96.14%
DT	78.63%	79.68%	78.99%	97.34%	96.81%	97.08%
	BI-RADS C					
	Before pre-processing			After pre-processing		
Classifier	Sensitivity	Specificity	Accuracy	Sensitivity	Specificity	Accuracy
KNN	70.62%	72.99%	71.74%	89.57%	88.87%	89.13%
SVM	98.09%	54.79%	76.36%	97.64%	82.54%	90.08%
DT	69.06%	71.51%	70.24%	90.89%	88.26%	89.54%
	BI-RADS D					
	Before pre-processing			After pre-processing		
Classifier	Sensitivity	Specificity	Accuracy	Sensitivity	Specificity	Accuracy
KNN	53.77%	51.62%	52.83%	68.97%	70.08%	69.67%
SVM	57.74%	64.86%	61.17%	83.34%	75.32%	79.33%
DT	54.86%	51.86%	53.33%	72.34%	69.59%	71%

**Table 12:** ROI’s Classification results of all BI-RADS classes using the generalized fractal dimension method

Features	Multifractals			Multifractals and GLCM		
Classifier	Sens (%)	Spec (%)	Acc (%)	Sens (%)	Spec (%)	Acc (%)
KNN	96.68	97.24	96.97	97.83	96.87	97.34
**SVM**	**98.04**	**97.17**	**97.61**	**98.66**	**97.77**	**98.20**
DT	97.37	97.39	97.38	97.53	97.03	97.27

*Acc* accuracy, *Sens* sensitivity, *Spec* specificity

**Table 13:** Comparison of classification results with state-of-the-art methods

Method	Database (no. images)	No. ROIs	Feature type (number)	Classifier	Acc (%)	Sens (%)	Spec (%)
[19]	BCDR (176)	Normal: 96 MC: 96 Total: 192	Statistical, interest points, interest corners (50)	Random forest	95.83	96.84	95.09
[20]	DDSM (100)	Normal: 10 MC: 10 Total: 110	Physical characteristics (2)	–-	99	99	100
[21]	IRMA (–-)	Normal: 932 MC: 688 Total: 1620	Rotation invariant local frequency magnitude descriptors	SVM	91.10	98.04	81.17
[22]	Mini-MIAS (–-)	Normal: 208 Abnormal:112 Total: 330	ResNet features	ResNet	95.91	–-	–-
[23]	DDSM (219)	Normal:124 MC:95 Total: 219	Statistical features (6)	SVM, LDA, MLPNN	90.9	98.4	81.3
[24]	BCDR (–-)	Normal: 130 MC: 130 Total: 260	Interest points, interest corners (2)	Random forest	97.31	94.62	100
**Proposed approach**	**INbreast (308)**	**Normal: 1344** **MC: 1344** **Total: 2688**	**Multifractals and GLCM (9)**	**SVM**	**98.20**	**98.66**	**97.77**

*Acc* accuracy, *Sens* sensitivity, *Spec* specificity, *Abnormal* masse + MC

The classification results obtained after the pre-processing step are very striking and persuasive, which proves that the proposed method for mammogram pre-processing is effective in enhancing the contrast of MCs by reducing the effect of breast density.

By comparing the two methods, the MFDMA method is not only complicated in its implementation where several parameters need to be adjusted and takes more computation time, but also does not give good classification results compared to the generalized fractal dimensions method.

In most research, the analyzed ROIs of MCs are ROIs where the MCs (clusters in general) are very clear and discriminated from the surrounding tissue. Also, critical cases where MCs are invisible and masked by the high breast density (BI-RADS C and D) are not analyzed in their studies. In our work, it should be noted that the analyzed ROIs contain only one MC, so, if we take ROIs containing more than one MC (cluster for example) or a mass, the result would be better since the multifractal spectrum was sensitive to one MC.

The BI-RADS classification classifies the mammogram according to the density of the breast tissue from the weakest to the densest. The denser the tissue, the more difficult detection is because breast density masks lesions, especially small lesions such as MCs. In this paper, all BI-RADS cases have been taken into consideration (BI-RADS A, B, C, and D). The classification accuracy obtained for classes A, B, and C exceeds 97%; for class D, the obtained accuracy of 93.33% is low compared to other classes, but it remains a promising result in detecting MCs. Grouping all cases, the classification accuracy reached 98% which is a very satisfactory rate compared with the literature.

The proposed algorithm could be efficient for other databases, such as BCDR or DDSM. The pre-processing step may not work the same way, since they do not have the same image quality, further filtering steps are required, but multifractal analysis is still a powerful tool for singularity analysis.

## Conclusion

In this paper, we have proposed an aided-system for the detection of MCs and classification of mammographic ROIs into normal or abnormal ROIs based on multifractal features. The proposed approach is based on multifractal analysis where the multifractal measures were used to enhance the contrast of the MCs, and then the multifractal spectrum was computed to extract the multifractal attributes used for the classification of the ROIs.

Generally, multifractal analysis does not require a pre-processing step, because it is a point analysis, which measures the local regularity and studies its variation from one point to another. In this paper, the pre-processing step was necessary for a better characterization and classification of ROI. A brief comparison of two methods of spectrum computation was made where the generalized fractal dimension method gave the best results. The combination between the multifractal features and the GLCM features gives a better result of classification where the SVM classifier was the most efficient. In comparison with the literature, the proposed system has given very satisfactory and promising results. The particularity of our work lies in the fact that all cases of breast density have been taken into consideration, which has not been done in the literature. On the other hand, the analyzed abnormal ROIs in our study contained only one MC, i.e., the spectrum was sensitive to one MC.

Finally, if this approach has been effective for the detection of individual MCs (ROIs containing one MC), certainly, it will be effective for the detection and classification of ROIs containing other abnormalities such as clusters of MCs or masses. Also, this approach could be used for the discrimination and classification of benign and malignant breast abnormalities.

## Data Availability

The INbreast dataset used in this work was assigned by the manager with a signed transfer agreement.

## Code Availability

Some matlab codes used in this work are available in Matlab toolboxes, and some others programmed by the authors are not available, they are personal.
